# Phrenology and Dentistry

**Published:** 1871-07

**Authors:** H. L. Ambler


### PHRENOLOGY AND DENTISTRY.

BY H. L. AMBLER, D. D. S., M. D.

Read before the Forest City Society of Dental Surgeons.

"Phrenology, is a system of mental philosophy founded on the physiology of the brain. It treats of mind, as we know it in this mortal life, associated with matter, and acting through material instruments." It is established on the broad and immovable basis of the constitution of man, and when practically applied, enables us from the size and shape of the head, in connection with observations on the body, to judge of the natural tendencies and capabilities of the individual. It teaches us to cultivate and develop all the higher qualities of mind and heart; to make the most of ourselves and our opportunities. For the more we know of anatomy, chemistry, physiology, and all other sciences, the better will we be able to conform ourselves or others, to a high standard of excellence, both moral and physical. The convolutions of the brain are the mind's revolvings, and are here represented in beautiful moving spirals; and the subtile insinuation of thought, whose path is through all things, issues with power from the form of these graceful cerebral screws. They print their shape and make themselves room on the inside of the skull, and are the most irresistible things in the human world. "In size, texture, and configuration, the brain partakes of the nature of the soul, and as we find the brain, so will we find in harmony with it the cranium, from which we read in general forms, or special elevations and depressions, the intellectual and moral character of man. The skulls of nations and races differ widely in form, and these differences are found to correspond with known national characteristics. The brain is the organ of the mind, every faculty of which has a special organ in the brain; thus every mental affection must be accompanied by a corresponding state of its separate organ in the brain, and the manifestations of mind will bear a strict relation in power and variety, to the size, strength and quality, of its instrument. Having stated the main principles which we are to consider, let us enquire what special development of organs are necessary to constitute a successful dental surgeon? We shall first notice the perceptive faculties. The organ of individuality, is situated in the center of the lower part of the forehead immediately above the base of the nose; it tends to observation, and leads to giving a specific form to all the ideas of the mind. The organ of form, is located at the internal angle of the orbit; it enables us to remember forms and objects of all kinds, and to judge well of symmetry and proportion. The organ of order is situated over the external angle of the eye; it gives preciseness, method, and is a co-worker with the reflective faculties in the conception of system and classification. The organ of destructiveness, is situated immediately above the ear, and when well developed, gives prominence to the center of the basilar region of the head; it gives us force and energy to overcome obstacles, to endure and inflict pain, as in surgical operations, to face trying things in our practice, to be resolute and not shrink from inflicting pain which is requisite to a cure. If necessary to cultivate this organ, do so by striving to remove or destroy whatever impedes your progress, as the removal of bone or tissue, sensitive dental caries, on extracting roots; do not be afraid of causing pain. The organ of constructiveness, is situated at the inferior outer part of the frontal bone, just above the spheno-temporal sutures; measure from the middle of the top of the ear, one inch upward, and then about $1\frac{3}{4}$ inches horizontally forward, and you locate this organ; it gives skill in manipulation, mechanical ingenuity, enables one to construct whatever his necessities or higher sentiments require, coming from the most simple artificial, to the finest gold operation, it is essential in making instruments, carving, or anything requiring manual nicety. This faculty is generally well developed in American heads, and when combined with good imitation, gives one the power to produce almost anything they have a pattern for, or have seen made, and they often contrive new ways of doing things, always using their instruments with skill. Such elements as these, combined with practice, will make a good operator. The organ of combativeness, is situated about $1\frac{1}{2}$ inches back from the top of the ear, corresponding with the inferior posterior angle of the parieteal bone, above and a little behind the mastoid process; it gives resolution, promptness, courage, and a will to overcome obstacles A considerable development is indispensable to great character, such as would enable the possessor to look undaunted on a contest for humanity or virtue's sake, and meet it without shrinking. This faculty tells us to face danger in any form, letting no difficulties discourage in carrying out plans, but be cool, never lose presence of mind, and strike boldly in a good cause. The physiognomical sign, is a marked enlargement of the neck below the organ; also prominence of the ridge of the nose is believed to be another sign. To cultivate this faculty, when deficient, we should seek to engage in mental discussions with those of our profession, whose ideas are somewhat different from our own; we should not shrink from, but rather court those operations which call out our highest powers of courage, resistance, and skill. The organ of cautiousness, is situated on the lateral posterior part of the head; by drawing a perpendicular line upward from the back part of the ear to where the head begins to round off, we strike upon this organ, which renders us judicious, prudent, watchful, guarding against danger, knowing it to be more difficult to sustain, than to acquire a good reputation; new undertakings are prosecuted carefully at first, anxious to anticipate any cause of trouble. Cautiousness may be aroused to activity by intellectual effort. A prudent and watchful policy should be adopted, observing where we have failed in our attempts before; we should be determined to remedy these defects, knowing that most of our misfortunes come from some fault of our own, or are the result of rashness; possibly we have not taken the time we feel we ought to have done in making preparation, before trying an operation, or may be we pass over a main point thinking it will answer. Those in whom this faculty is well developed, seldom break any article, and work with sharp instruments without cutting themselves; are judicious in making plans, rather deliberate, counting the cost before engaging in any enterprise. Nature intends that our own natural predilections should be somewhat of a guide in choosing a pursuit for life, and we claim that a good phrenologist will predict in advance whether a boy will succeed in our profession. He does it by consulting his organization and determining what are his tastes, tendencies and capacities, and instead of making a dry monotonous preacher, an indifferent lawyer, or a blundering dentist, he tells him he will succeed finely as a business man, machinist or farmer. There are only a few persons with such well balanced heads, that they would be successful in almost any undertaking. Self knowledge has been always considered the most important of all learning; then phrenology, which is the key to this knowledge must be of great moment, for it enables us to ascertain our own weak points and guard against them; also to cultivate those faculties which are deficient, and would otherwise lead us in a wrong direction, for by right cultivation do we turn them to paths of purity. Next to knowing ourselves, is to know our fellow creatures, those whom we come in contact with professionally, for much of our happiness and success depends upon our friendship for them, and the knowledge we have of their characters. Phrenology gives us success through an understanding of human nature, and tells us how to treat our patients, among whom we find all manner of organizations. In controlling the children we operate for, no one method will answer, but we find we are obliged, in order to succeed, to address ourselves to their mental organization, thus may we discipline and govern them in a reasonable manner, knowing that each faculty is normally good, we can proceed in a proper way to gain their confidence. Every faculty of the human mind is capable of being improved by culture, this fact is of practical importance, and will give encouragement to the faltering. If we wish to develop any part of the body for a certain feat of endurance or agility, we give it a training under proper circumstances, and by well adapted exercises; thus do we proceed with each or any faculty of the mind, applying the necessary culture, through its organ, the brain. Developing the lower faculties, and having them act in harmony with the higher, will contribute much to the happiness of man. It is necessary that one in our profession, should have a contented mind, for how well we know that emotions of grief induce a relaxation of the system, especially affecting digestion and secretion; thus do all conditions of body and mind, whether constitutional and permanent, or pathological and temporary, act and react upon each other. Hope and joy quicken the circulation, steady and brace the nerves, strengthen the muscles, and fit us for our best efforts. A combination of the *motive* and *mental* temperament is desirable, not having either predominate to excess, for it would have a tendency to lessen the symmetry and harmony both of body and mind, as one is influenced so much by the other, for in this development, we find our most active professional men, who are generally known by fair length of body and moderate fulness of limb. We find the most excitable, are those, having the vital and mental temperament greatly developed; being morbidly active in those whose nerves are disordered, and where the system has been the subject of abuse from alcoholic stimulation. Strive to guard against an excess of excitability, knowing that where all the faculties act harmoniously together, the real effectual strength of each is increased. Dental surgeons should have good health, for we are aware that from sound bodies, the great thoughts which have moved our profession and the world, have emanated. If the body be weak and sickly, we can no more think or study effectively, than we can wield the sledge hammer or follow the plow, much less manipulate in delicate operations, which require a certain tension of muscle, brain and nerve, which is taxing to the general system, in the highest degree. Respiration is one of the great functions of all physical life; as breath and life are identical, our vitality being in proportion to our respiration; and it really is quite true that "people die for want of breath," but it is their own fault, for they only *half* breathe, therefore they only *half* live. Cultivate this failing, by expanding the lungs slowly to their utmost capacity, many times daily in the fresh air. Digestion is effected by respiration and circulation; should the latter be deficient, the proper amount of blood is not sent to the stomach during digestion; and should the former be imperfect, the blood will lack strengthening power, as good blood is obtained from pure and well assimilated food, well oxygenated by breathing plenty of pure air; thus from good blood, circulation and respiration, we have built up structures of healthy muscle, nerve and brain. Let us not forget to take into consideration the *quality* of the *brain;* the quantity may be great and sufficient, but where it lacks firmness in texture. there will be no remarkable traits. Muscle to be the best, should be firm and tough, this would bring us to conclude, that the greatest minds are found, where there is a bulky compact brain, connected with a firm, densely knit, well constructed body. Yet animal power is not mind, but is that something which is required by the mind in this world; to manifest itself through a diseased state of the body, affects memory, reason, and philosophy; weakens the system generally, and those channels through which we were wont to receive pleasurable emotions, have with the thrill of an instant, electrified millions of nerves which spring to execute their perverted functions, wreaking on us the most terrible physical and mental torture.

